# Emerging Issues for the Public Health Laboratory^1^

**DOI:** 10.3201/eid1011.040797_13

**Published:** 2004-11

**Authors:** Patricia Somsel, David Warnock

**Affiliations:** * Michigan Department of Community Health, Lansing, Michigan, USA;; † Centers for Disease Control and Prevention, Atlanta, Georgia, USA

**Keywords:** public health labs, pathogen emergence, testing methods, security requirements, personnel shortages

U.S. public health laboratories face challenges from within and outside the system, including emergence of new pathogens, introduction of new testing methods, new security requirements, shortages of well-qualified personnel, and collaboration with new partners.

The public health system depends on hospital and commercial laboratories as major sources of reliable epidemiologic information. Thus, the current crisis in these laboratories is of great concern. The pressures come from the need to address emerging infectious diseases, detect antimicrobial resistance, and recognize potential agents of bioterrorism while updating procedures, practices, and facilities to meet new biosafety, biosecurity, confidentiality, and other regulations. Laboratories face a rising demand for services from an aging population at increasing risk for infectious diseases.

Clinical laboratories are receiving diminished revenues and facing increased productivity demands that result from downsizing, consolidation, and mergers. There is a shortage of qualified personnel, resulting from loss of senior staff because of retirement and difficulties in recruiting and retaining younger microbiologists. A solution to this crisis will require higher starting salaries, better tuition reimbursement, increased provision of distance learning for current staff, and increased test automation.

Bioscience laboratories are potential sources of threatening pathogens and toxins. Control of these materials is essential, but how this is achieved must be carefully considered and implemented. Potential threat agents can often be acquired from nonbioscience sources. Moreover, the nature of these materials makes their diversion difficult to prevent, and because many biological materials and technologies have dual uses, illegitimate activities can be very difficult to detect. Although many security experts believe that the most credible threat comes from persons with legitimate access to bioscience facilities, security at such facilities has largely been focused on protection against outside adversaries. Such facilities cannot be protected unless their staff understand and accept the need for security measures.

To adequately protect collections of virulent biologic agents, those responsible for the design of biosecurity systems must understand biologic materials and research and have the active involvement of laboratory scientists. Since risk will always exist and every asset cannot be protected against every threat, distinguishing between acceptable and unacceptable risks is imperative. Facilities should conduct an agent-based, security-risk assessment to ensure that protection of their assets is proportional to the risk for theft or sabotage of those assets.

The list of potential human health, animal, and agricultural threat agents is extensive. Areas at risk include not only public health and well-being, but economic well-being, public trust, consumer confidence, and the national infrastructure.

Forensic science involves applying scientific procedures to the investigation of both criminal and civil legal matters. The principal questions that microbial forensics sets out to answer are the following: What is the agent? Was the event intentional? Was the pathogen engineered? Where did the pathogen come from? and Who committed the crime? The manner in which forensic evidence is generated is critical if it is to be admissible in court. To assist law enforcement, the Scientific Working Group for Microbial Genetics and Forensics has been established. This group has identified research needs for methods to identify and type threat agents. It has established quality management guidelines for laboratories, with the goal of promoting development of forensic methods that are rigorous and scientifically valid.

Recent reports from the Institute of Medicine (*1*) and others recognize that the public health laboratory system has many components. The challenge presented by emerging and reemerging infectious diseases, whether these be old microbes with new scenarios (e.g., *Bacillus anthracis*), new microbes (e.g., severe acute respiratory syndrome), or old microbes with new resistance patterns (e.g., multidrug-resistant *Mycobacterium tuberculosis*), requires greater coordination between public health, clinical, and commercial microbiology laboratories. Each segment produces unique, yet overlapping, data essential to the nation's health. Essential to good coordination is communication, which can be enhanced by joint participation in meetings, collaborative studies, training opportunities, cross-cutting committees, and service on regional or national advisory boards.
